# Ageing and microvasculature

**DOI:** 10.1186/2045-824X-6-19

**Published:** 2014-09-16

**Authors:** Maria Giovanna Scioli, Alessandra Bielli, Gaetano Arcuri, Amedeo Ferlosio, Augusto Orlandi

**Affiliations:** 1 Department of Biomedicine and Prevention, Institute of Anatomic Pathology, Tor Vergata University, Via Montpellier, Rome 00133, Italy

**Keywords:** Endothelial cells, Smooth muscle cells, Endothelial dysfunction, Nitric oxide, Vascular remodelling, Organ-specific ageing

## Abstract

A decline in the function of the microvasculature occurs with ageing. An impairment of endothelial properties represents a main aspect of age-related microvascular alterations. Endothelial dysfunction manifests itself through a reduced angiogenic capacity, an aberrant expression of adhesion molecules and an impaired vasodilatory function. Increased expression of adhesion molecules amplifies the interaction with circulating factors and inflammatory cells. The latter occurs in both conduit arteries and resistance arterioles. Age-related impaired function also associates with phenotypic alterations of microvascular cells, such as endothelial cells, smooth muscle cells and pericytes. Age-related morphological changes are in most of cases organ-specific and include microvascular wall thickening and collagen deposition that affect the basement membrane, with the consequent perivascular fibrosis. Data from experimental models indicate that decreased nitric oxide (NO) bioavailability, caused by impaired eNOS activity and NO inactivation, is one of the causes responsible for age-related microvascular endothelial dysfunction. Consequently, vasodilatory responses decline with age in coronary, skeletal, cerebral and vascular beds. Several therapeutic attempts have been suggested to improve microvascular function in age-related end-organ failure, and include the classic anti-atherosclerotic and anti-ischemic treatments, and also new innovative strategies. Change of life style, antioxidant regimens and anti-inflammatory treatments gave the most promising results. Research efforts should persist to fully elucidate the biomolecular basis of age-related microvascular dysfunction in order to better support new therapeutic strategies aimed to improve quality of life and to reduce morbidity and mortality among the elderly patients.

## Introduction

**Figure 1:**
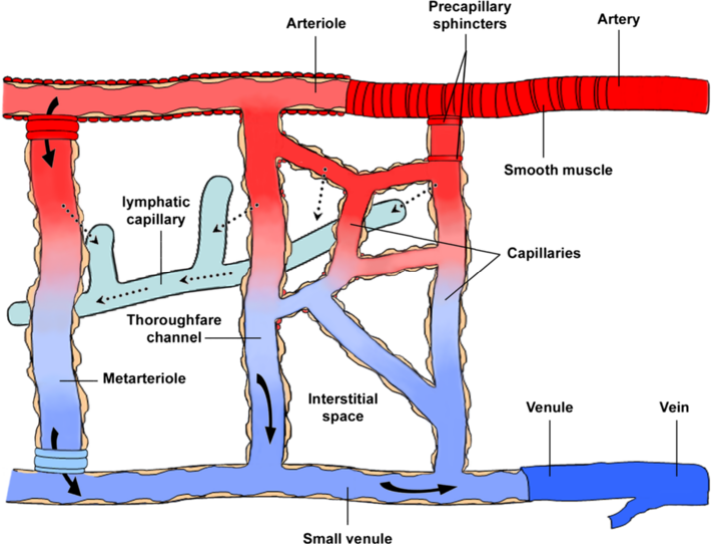
**Schematic representation of microcirculation components.** The microcirculation is a network of small blood vessels, including arterioles, venules and capillaries. Blood flows from the arteries into the arterioles and then pass into the venules across true capillaries or throughfare channels and metarterioles (arteriovenous bypass). The precapillary sphincter, made of smooth muscle cells, controls blood flow into the true capillaries. As blood travels through the capillaries, plasma proteins and fluid enter the interstitial space according to hydrostatic and osmotic pressure gradients. Most of the fluid is reabsorbed into the post-capillary venules, while a fraction enters to the lymphatic circulation for its return to the blood circulation.

Vascular ageing is associated with both structural and functional changes that can take place at the level of the endothelium, vascular smooth muscle cells and the extracellular matrix of blood vessels [1]. Age, hypertension, diabetes, smoking and plasma low density lipoprotein cholesterol level are determinant risks of arterial stiffness [2,3]. A relevant age-related vascular change is a progressive myointimal thickening [4,5]. Similarly to that observed in large vessels, age-related increase of microvascular tone leads to a progressive myogenic hypertrophic remodelling of small arteries, due to the increased distending pressure acting perpendicularly on the vascular wall [6]. Microvascular alterations play an important role in ageing-associated end-organ damage [7]. In fact, microcirculation provides the interface for tissue delivery of oxygen and nutrients, removal of waste products and carbon dioxide, transvascular exchange and fluid economy [8]. Therefore, cell survival depends on adequate microvascular perfusion [8]. The architecture and the biophysical behavior of flowing blood strongly influence microvascular function. Morphologically, the microcirculation is constituted from vessels <300 μm in diameter [8]. Therefore, it includes arterioles, capillaries, and venules (Figure 1). Alternatively, a physiological definition based on vessel function rather than diameter or structure has been proposed [9]. By this definition, vessels that respond to an increase of pressure by a myogenic reduction in lumen diameter are considered part of the microcirculation [9]. Consequently, besides endothelial cells, also vascular smooth muscle cells (VSMCs) and pericytes must be included in the microvascular cell population. Although the primary function is to optimise the nutrient and oxygen supply, microcirculation is relevant in order to avoid large hydrostatic pressure fluctuations causing disturbances in capillary exchange and an overall peripheral vascular resistance [10]. An important role in regulating tissue fluid balance and in maintaining osmotic and hydrostatic pressures is played by the lymphatic system (Figure 1), that comprises a one-way transport for fluid and proteins by collecting them from the interstitial space and returning them to the blood circulation [11]. This review focuses the attention on the biomolecular and pathophysiological mechanisms underlying age-related microvascular alterations and the importance of new therapies to prevent end-organ damage associated with microvascular dysfunction.

### Biomolecular mechanisms involved in age-related microvascular dysfunction

#### Reactive oxygen species and oxidative stress

The primary mechanism involved in ageing-associated microvascular dysfunction is the oxidative stress, a state in which the generation of reactive oxygen species (ROS) exceeds the antioxidant defense systems, resulting in cellular dysfunction and apoptosis [12]. Physiologically, ROS are involved both in the maintenance of steady vessel wall conditions and in the vascular response to altered flow or pressure settings [12]. Vascular cells comprise different sources of ROS, including enzymatic activity of NAD(P)H oxidase, xanthine oxidase (XO), uncoupled endothelial nitric oxide (NO) synthase (eNOS), cytochrome P450 and the mitochondrial respiratory chain [13-15]. The main component of ROS is the superoxide anion (O2-), which for its high cytotoxic activity is transformed quickly into hydrogen peroxide (H2O2) by superoxide dismutase (SOD). The H2O2 is transformed in H2O by two enzymes, catalase and glutathione peroxidase (GPx) [16].

As reported, with ageing H2O2 production is enhanced [17], leading to the increase of mitochondrial H2O2 and O2- generation, cumulative DNA damage and cellular senescence [18-20]. Moreover, mitochondria are not only targets for ROS but also significant sources of ROS, which under certain conditions may stimulate NAD(P)H oxidases [12]. In fact, many studies demonstrated the principal role of NAD(P)H oxidase activity in aged-mediated ROS generation in mouse models [21-23] and the improvement of endothelial function by the inhibition of NAD(P)H oxidase or scavenging of O2- [24,25]. In particular, it has been reported that NAD(P)H oxidase 4 is involved in O2- formation and cellular senescence in ageing, and its inhibition counteracted oxidative stress in pulmonary and kidney arteries of aged rats, as well as in lungs of aged mice [26-28].

### Nitric oxide

In mammals, nitric oxide (NO) is produced by a family of enzymes, named nitric oxide synthases (NOSs), that catalyse the production NO from L-arginine. NO is an important cellular signalling molecule that regulates vasodilatation, insulin secretion, airway tone, and peristalsis, and is involved in angiogenesis and neural development [29]. The family of enzymes NOS comprises three isoforms: neuronal NOS (nNOS/NOS1), inducible NOS (iNOS/NOS2) and endothelial NOS (eNOS/NOS3) [29] eNOS constitutively produces NO in endothelial cells and physiologically contributes to the control of vascular tone. Instead iNOS is activated by bacterial lipopolysaccharide, cytokines, and other inflammatory agents, determining an abnormal production of NO. Due to its affinity to protein-bound iron, NO can inhibit key enzymes that contain iron in their catalytic centers. These include iron–sulfur cluster-dependent enzymes (complexes I and II) involved in mitochondrial electron transport, ribonucleotide reductase (the rate-limiting enzyme in DNA replication), and *cis*-aconitase (a key enzyme in the citric acid cycle) [29].

As discussed above, microvascular dysfunction is mainly induced by the over-production and release of O2-, which cause NO breakdown. In fact, NO inactivation is due to its reaction with O2- to form the potent oxidant peroxynitrite (ONOO^−^) [30]. This compound can cause oxidative damage, nitration, and S-nitrosylation of biomolecules including proteins, lipids, and DNA single-strand breakage following the poly-ADP-ribose polymerase (PARP) activation [31-33]. The increase of nitration was demonstrated in the sarcoplasmic reticular Ca-ATPase isolated from the skeletal muscle of old rats [34]. The scavenging of NO by O2- was also demonstrated in coronary microvascular endothelial cells of old rats, in which the reduction of eNOS expression was accompanied with an increased O2- production and attenuated vasodilator responses [35]. Coronary arterioles of aged rats displayed an increased iNOS activity and ONOO^−^ production, as well as a decreased eNOS expression [36]. The same alterations have been also described in elderly [36].

Moreover, oxidative stress can convert eNOS from a NO-producing enzyme to an enzyme that generates O2-. This process is named eNOS uncoupling. Mechanisms implicated in eNOS uncoupling include oxidation of the critical NOS cofactor BH_4_, depletion of L-arginine, and accumulation of endogenous methylarginines [29].

### Age-related signal alterations in vascular cells

It has been demonstrated that endothelin-1 and angiotensin II (potent vasoconstrictors) pathways are involved in age-related endothelial oxidative stress [18]. In particular, ageing induced endothelin-1 overexpression, resulting in vascular remodelling and endothelial dysfunction in mice [37]. In addition, it has been reported the involvement of endothelin-1 in eNOS downregulation in pulmonary artery endothelial cells of fetal porcine [38]. As concerning angiotensin II, it has been documented that in ageing its overexpression caused vascular senescence by mitochondrial and NADPH-dependent superoxide generation [18]. This mechanism was attenuated by mitochondrial electron transport chain or angiotensin type 1 receptor inhibitors [39,40]. Moreover, the infusion in rats of angiotensin II induced microvascular lesions in various vascular beds that resemble arteriolosclerosis [41]. The blocking of nitric oxide synthesis also induced renal microvascular disease [42].

It is well known that angiogenesis and wound healing are reduced with ageing [43]. In fact, vascular endothelial growth factor (VEGF)-induced angiogenesis is attenuated in aged rats and rabbits [44,45]. In aged mice and in cultured human microvascular endothelial cells aged by progressive passaging, the expression of the tissue inhibitor of metalloproteinase-2 (TIMP-2) is increased [46], and correlated with an attenuated capacity of endothelial cells to degrade extracellular matrix, a process required for angiogenesis [46].

**Figure 2:**
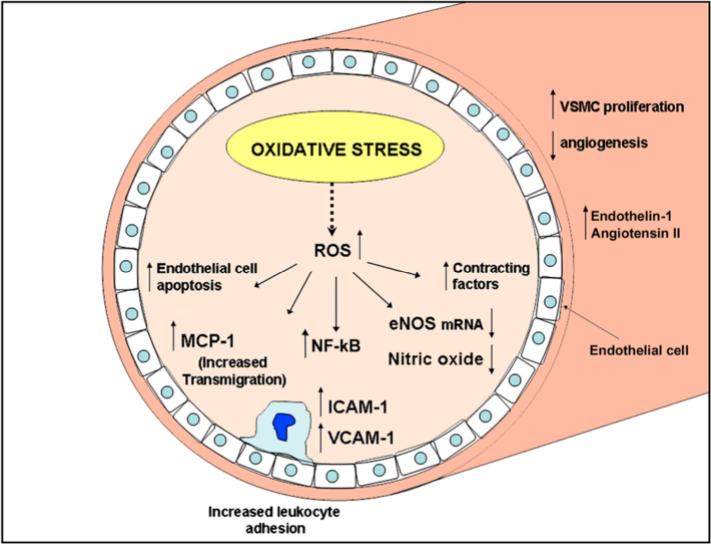
**Schematic representation of biomolecular changes in age-related microvascular dysfunction.** Oxidative stress plays a pivotal role in endothelial and myocitic impaired function.

Taken together these findings suggest the existence of a complex biomolecular mechanism involved in age-related vascular dysfunction that leads to oxidative stress, vascular remodelling and endothelial dysfunction. This altered signalling, in endothelial cells, causes the activation of NF-kB and a consequent abnormal gene transcription, including the enhancement of cellular adhesion molecule expression, such as intercellular adhesion molecule-1 (ICAM-1), vascular cell adhesion molecule-1 (VCAM-1), E-selectin, and inflammatory cytokine secretion [47-49]. This process determines the leukocyte recruitment and extravasation as also demonstrated in the vascular wall of aged rabbits [50]. A schematic representation of age-related biomolecular alterations in microcirculation is reported in Figure 2.

### Structural and functional microvascular alterations involved in ageing

#### Arteriolosclerosis

**Figure 3:**
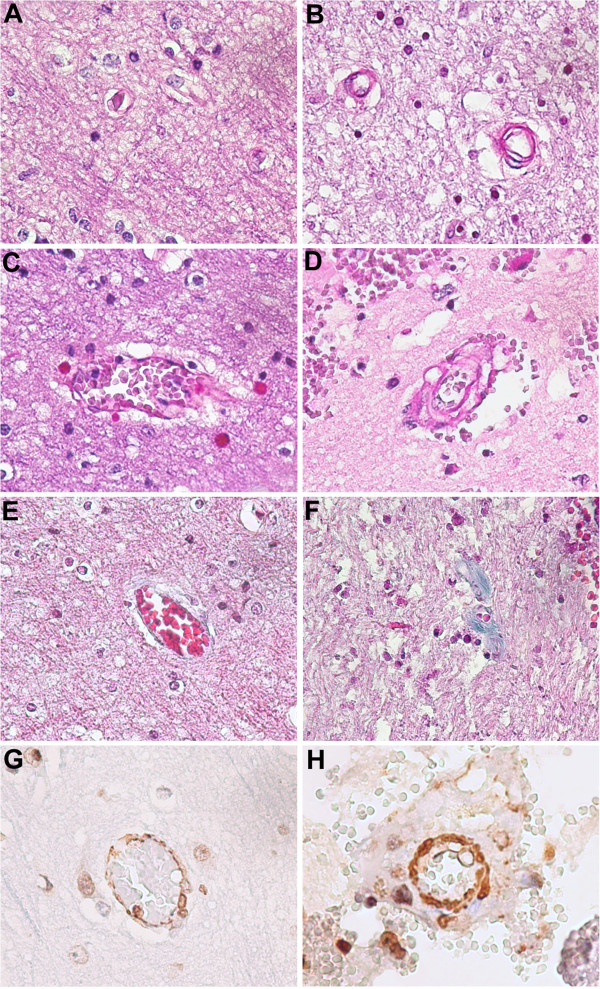
**Age-related changes of brain microvasculature.** Post-mortem (myocardial acute infarction) histology studies on paraffin-embedded sections (5 μm thick) of formalin-fixed cerebral tissue. PAS staining of human brain gray matter, showing normal capillaries and arterioles in a young **(A,C)** compared to concentrically thickened microvessels in an aged man **(B,D)** mostly due to hyalinization (pink staining). Masson's trichrome staining shows normal microvessel in a young **(E)** and perivascular deposition of collagen (blue staining) around capillaries in an aged man **(F)**. Immunohistochimical analysis for α-SMA shows normal arteriole in a young **(G)** and concentrically thickened arteriole due to an altered proliferation of smooth muscle cells in an aged man **(H)**. Magnification 40×.

Microvascular disease is also referred as an impairment of flow-induced dilatation of arterioles, defined arteriolosclerosis [51]. Arteriolosclerosis is due to stiffening, with loss of elasticity, of arterioles and must be distinguished from arteriosclerosis, a hardening with loss of elasticity of medium or large arteries, and from atherosclerosis, a stiffening of an artery specifically due to an atheromatous plaque. Arteriolosclerosis is characterised by intimal thickening, vascular smooth muscle cell proliferation, and extracellular matrix deposition, resulting in an increased media-to-lumen ratio, and later by the replacement of the vascular smooth muscle cells by areas of fibrosis and cell loss [51]. Consequently, arteriolosclerosis may have a key role in mediating the development of chronic kidney disease, vascular dementia, stroke and coronary heart disease [51]. Hyaline arteriolosclerosis refers to a thickening of the wall of arterioles by the deposition of homogeneous pink hyaline material and can involve multiple organs, including brain (Figure 3). Structural and functional alterations described above involve all microvascular components, including endothelial cells, pericytes and smooth muscle cells. Below are summarised the principal features of age-related microvascular cell changes.

### Endothelial cells

As a consequence of the alteration in the expression and/or activity of eNOS, upregulation of iNOS, and increased formation of ROS and ONOO^−^, endothelial cells undergo to cumulative DNA damage that promotes senescence and apoptosis [52]. As described above, the age-related decline of endothelial function becomes manifest through a reduced regenerative and angiogenic capacity, and an altered expression of adhesion molecules regulating the interaction of circulating factors with immune system cells [53,54].

The attenuated capacity of the endothelium to regenerate is partially a consequence of an impaired secretion of and/or sensitivity to growth factors [55]. Recently, the regeneration of the endothelium by bone marrow-derived circulating progenitor cells has gained particular attention, because the number of circulating endothelial progenitor cells (EPCs) decreases with age and is thought to reflect the attenuated mobilization of these cells from the bone marrow [56]. Moreover, EPCs from older subjects have a reduced capacity to engraft [57]. Some studies suggest that the regenerated endothelium is functionally impaired [57] and exhibits an increased uptake of modified low-density lipoprotein (LDL) and decreased NO production [58]. For example, as documented in aged rats, important structural changes of brain capillaries were found: thickening of the basal lamina and the thinning of endothelial cells [59]. Some suggest that this phenomenon is due to a loss of endothelial cells together with a lengthening of the remaining ones to allow nutrients to diffuse [60]. Mophological alteration of aged endothelium was observed also in sinusoids of human aged liver, where thickening of the sinusoidal endothelium was associated with the deposition of basal lamina and collagen [61]. In the kidney of aged rats the number of proliferating endothelial cells was decreased compared with young rats. In addition, VEGF expression strongly decreased with ageing in the endothelium of the outer and inner medulla, suggesting a reduced angiogenic activity [62].

### Smooth muscle cells and pericytes

As discussed above, in ageing, upregulation of pro-oxidants and downregulation of antioxidants results in an imbalance leading to ROS increase [63-65] and to the development of vascular dysfunction in both animal models and in humans [66]. In old rats, a significant increase in O2- was observed in the vascular wall [67], and was associated with an increase in NAD(P)H oxidase activity [36,64,68-70]. It has been also reported that Angiotensin II pathway plays an important role in age-related smooth muscle cell oxidative stress by eliciting NAD(P)H oxidase activity [71]. In fact, Angiotensin II stimulation induced the NAD(P)H oxidase-dependent O2- production, stimulating NF-κB signalling in senescent VSMCs [72]. Similarly to endothelial cells, VSMCs of old rats in response to cytokines showed higher ICAM-1 level compared with newborn rats [73]. VSMCs can also induce the activity of iNOS through the NF-kB pathway under inflammatory conditions [64], as also reported in aged Macaca mulatta, rats [64,74] and mice [75]. As already reported, vascular ageing is also associated with a progressively reduced NO bioavailability. Since VSMCs are important targets for endothelium-derived NO, this reduction causes an impairment of endothelium-dependent vasodilation [76]. In addition, the *in vitro* response of VSMCs to NO and β-adrenoreceptor stimulation is decreased by ageing, and such changes may contribute to impairment of endothelium-independent vasodilation in the elderly [76,77]. Consequently to age-related oxidative stress and impaired signalling transduction, VSMCs undergo to phenotypic alteration, proliferation, migration, dedifferentiation and extracellular matrix remodelling, as reported in coronary resistance arterioles of old rats [5]. The series of events lead to increased vessel wall thickness, inflammation, and vulnerability to the development of vascular dysfunction [64,78]. VSMCs lose their specialised or differentiated properties and become proliferative and highly motile [5,79]. Extracellular matrix reorganization occurs with ageing, such as collagen increase and elastin fragmentation [80]. These changes in the relative content and organisation of collagen and elastin result in increased fibrosis and contribute to the stiffening of the vascular wall [81]. It may be due to alternative signal transduction pathways revealed by the ability of the older cells to respond to inhibitors, such as transforming growth factor-β1, or to altered interactions with the extracellular matrix resulting from age-associated shifts in integrin expression [54]. Both b1 integrin, adhesive interactions with fibronectin and α-smooth muscle actin (α-SMA) are also major players in VSMC stiffening [82].

Pericytes, the mural cells on capillaries, play an important role in vessel stabilisation, by regulating endothelial cell proliferation and preventing capillary withdrawal [83-85]. Alterations in these cells with ageing also might contribute to the development of age-related morphological and physiological abnormalities of the microvasculature. In fact, microvascular ageing is characterised by changes in peripheral capillaries, including vessel broadening, and thickening of the basement membrane, as well as altered length and orientation of desmin filaments in pericytes [86]. These changes can determine a reduced pericyte–endothelial cell contact, destabilisating capillaries [86]. In addition, a reduction in pericyte number in aged capillaries was also reported [87]. In the brain capillaries of elderly the decrease in pericyte coverage was reported [88]. It has been also documented that in the retina of old rats, ageing induced the broadening of peripheral capillaries and terminal venules, as well as thickening of basement membranes [86]. In the retina of old rats was reported a shift from a pericyte phenotype toward an arteriolar smooth muscle cell–like phenotype. It was associated with an increase in calponin labelling of arterioles, thickness of basement membranes, and increased focal adhesions in arteriolar walls [86]. Moreover, in skeletal muscle of old mice, the muscular regenerative capacity of pericytes is limited, and they produce collagen and contribute to fibrous tissue depositing [89].

### Lymphatic vessel alterations

Lymphatic system begins when the plasma fluid and proteins, that are forced out by arterial capillaries into the interstitial space (Figure 1), are collected into the lymphatic capillaries, which are freely permeable to macromolecules [90]. So, the main function of lymphatic system is to maintain osmotic and hydrostatic pressures within the tissue space. It consists of capillaries (10-60 μm in diameter) that drain lymph into the collecting vessels that contain also smooth muscle. The fluid pass through several clusters of lymph nodes and then into larger trunks, which in turn lead into the ducts, that return lymph back into the bloodstream [11].

Spontaneous contractions of smooth muscle cells in the wall of lymphatic vessels are necessary to maintain effective lymph flow whereas proper functioning of lymphatic endothelial cells is necessary to regulate lymphatic contractility [91]. The basic self-regulatory mechanisms controlling lymph flow in lymphatic vessels is realised through the sensitivity of their muscle cells to levels of stretch and of their endothelial cells to levels of the shear stress [91]. Nitric oxide plays an important functional role in coordinating the lymphatic contractile cycle [92] and in fine tuning lymphatic contractions to different levels of basal luminal flow [93]. Zhdanov and Zerbino reported ageing-related changes in morphology of various human lymphatic networks in the early 1960s [90,94,95]. They observed a reduction in the number of lymphatic capillaries (nonmuscular initial lymphatics) through all of the body and the presence of specific “varicose bulges,” which exist in muscular lymphatic vessels. It has also been reported that aged thoracic duct showed signs of lipid accumulation, thickening, and fibrosis [90,96].

Recently, some authors reported changes in orientation and investiture of muscle cells in mesenteric lymphatic vessels in aged rats [90,91]. It has been postulated that in elderly the decrease of accessory muscle elements surrounding lymphatic valve may limit the ability of lymphatic vessels to adapt their contractility to various preload/afterload challenges with subsequent formation of lymph stasis and potential spread of pathogens and immune cells in direction opposite to the direction of the normal lymph flow [90]. In addition, the thin-walled low muscle cells investiture zones in aged rats may be transformed to aneurysm-like formations “varicose bulges”, which can be ideal places for formation of low-velocity turbulent lymph flow and accumulation of various molecules, pathogens, and cancer cells [90]. Some studies reported a reduced lymph flow in aged animals in vivo [97,98]. Ageing severely altered contractility of the toracic duct through weakening of lymphatic contractions and complete depletion of their shear/nitric oxide (NO)-dependent regulation [98]. It has been demonstrated that ageing severely altered NO-dependent regulation of thoracic duct contractions with an impaired eNOS function and an ageing-associated shear-independent NO release in the duct due to iNOS activation [98]. Non-specific nitric oxide synthase (NOS) blockade restored the contraction [98]. These findings provided functional consequences of ageing in lymphatic contractility and the dysfunctional responses of smooth muscle cells and endothelium in ageing-induced alterations [98].

### Age-related changes of end-organ microvasculature

As a consequence of the age-related alterations in the expression and/or activity of eNOS, upregulation of iNOS, increased formation of ROS and ONOO-, and extracellular matrix remodelling, vasodilatory function is impaired and an excessive capillary pressure with consequent hyperfiltration, protein leakage, edema formation and tissue damage occur. In small arteries and arterioles, which have a relative higher wall thickness, changes in tone and circumferential shortening have an enhanced effect on lumen diameter, resulting in a blood flow decline in many organs [7]. We describe the main alterations that characterise the age-related end-organ damage.

### Brain

Cognitive dysfunction from lower perfusion and microvascular fibrohyalinosis is the most common type of microvascular damage in the elderly [99]. Atherosclerosis in elderly people also coincides with massive microvascular fibrosis, which contributes to the development of white matter lesions, myelin rarefaction or demyelination, gliosis, apoptosis and regressive astrocytic changes [99-101]. Thickening of small vessels was associated with diffuse white matter lesions in elderly [102]. Reduced pericyte–endothelial cell contact also occurs [86].

Brain arteriolosclerosis is a subtype of cerebrovascular pathology characterised by concentrically thickened arterioles due to an altered proliferation of smooth muscle cells and excessive extracellular matrix deposition [103], as also shown in our histological study (Figure 3). Cerebral amyloid angiopathy (CAA) is another microvascular pathology associated with ageing and results from deposition of β-amyloid in the media and adventitia of small arteries and capillaries of the leptomeninges and cerebral cortex and is a major cause of lobar intracerebral hemorrhage and cognitive impairment in the elderly [104]. CAA is present in nearly all brains with Alzheimer disease, suggesting a common β-amyloid-based pathogenesis for these diseases. However, despite the close molecular relationship between the two diseases, CAA remains a clinically distinct entity from Alzheimer disease [104]. The accelerated β-amyloid vascular deposition in CAA seems to be caused by a transcriptional deregulation of the lipoprotein receptor LRP in VSMCs due to overexpression of the transcription factors: serum response factor (SRF) and myocardin [105]. In addition, SRF and myocardin may also regulate contractile proteins in VSMCs, thus altering normal vessel physiology [106].

### Liver

Age-related changes in the human hepatic sinusoidal endothelium, termed pseudocapillarisation, have been recently described and they contribute to the impairment of hepatic function [107]. Blood clearance of a variety of waste macromolecules takes place in liver sinusoidal endothelial cells (SECs) [108]. These cells are unique endothelial cells in both their architecture and their function. The sinusoids are the exchange vessels of the liver, and the SECs are distinguished by extensive fenestrations organized into sieve plates, a lack of a basement membrane, and low junctional expression of CD31 [108]. The SEC architecture, including open fenestrations and weak junctional association between cells, provides a dynamic filtration system with low perfusion pressure that enables nutrients and macromolecular waste to pass freely to hepatocytes for efficient metabolism [108]. The maintenance of SEC phenotype is a critical process that requires both autocrine and paracrine cell signalling [108]. Recent studies indicate that fenestrations are maintained by constitutive VEGF-stimulated NO generation in SECs and surrounding cells [109]. In response to ageing [110], SECs dedifferentiate into a more regular endothelium, hence the term capillarisation or pseudocapillarisation. The hallmarks of capillarisation are SEC defenestration, development of a laminin-rich basement membrane, junctional expression of CD31 and protein nitration, in a mechanism involving NAD(P)H oxidase–generated ROS [108]. In addition, sinusoidal stellate cells are also induced to overexpress a laminin and collagen matrix that contributes to fibrosis [111].

**Figure 4:**
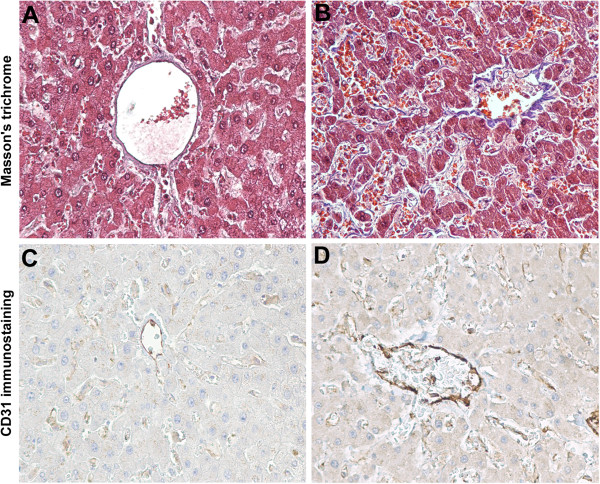
**Microscopic aspects of human liver pseudocapillarisation.** Post-mortem (myocardial acute infarction) histology studies on paraffin-embedded sections (5 μm thick) of formalin-fixed liver tissue. Masson's trichrome staining shows the central vein and pericentral hepatocytes of young **(A)** and old liver **(B)** with perisinusoidal collagen deposition (blue staining). CD31 immunostaining of young **(C)** and old liver **(D)** with an increased sinusoidal protein expression. Magnification 20×.

In autoptic studies of older human subjects, independently from the presence of systemic diseases or hepatic pathologies, pseudocapillarisation occurs from increased peri-sinusoidal expression of von Willebrand’s factor, CD31 and collagen I and IV, resulting in a thickening and defenestration of the liver sinusoidal endothelium and deposition of basal lamina in the extracellular space of Disse [61,107], as also shown in our histological study (Figure 4). In addition, it has been reported an endothelial upregulation of ICAM-1 [61]. Transmission electron microscopy study revealed a significant age-related thickening of the sinusoidal endothelium, with loss of fenestrations [61]. Loss of fenestrations leads to impaired transfer of lipoproteins from blood to hepatocytes. This provides a mechanism for impaired chylomicron remnant clearance and postprandial hyperlipidemia associated with old age [112].

### Heart

Ageing is also associated with functional changes of the coronary microvasculature [113]. An important mechanism that contribute to the local regulation of myocardial blood flow is the flow (shear stress)–induced NO mediated dilatation of small coronary arteries and arterioles [114]; so ageing, that impairs NO synthesis/release in the endothelium (as described above), determines a vasodilatory dysfunction also in rat coronary arterioles [115]. It was also reported an increased breakdown of NO due to an augmented arteriolar production of O2- [116]. Moreover, in isolated coronary arterioles of old rats, with an impaired flow-induced dilatation, O2- and ONOO- production increased both in endothelial and VSMCs [36]. In addition, eNOS and SOD activity were impaired, whereas NAD(P)H oxidase and iNOS were upregulated. [36]. Aged human and rabbit small coronary vessels show a marked increase of myocardial interstitial collagen, with α-SMA and TGFβ-1 negative fibroblasts and VCAM-1 positive microvessels without macrophages [117,118]; these findings support the close link between endothelial dysfunction and age-related fibrosis [117,118]. The impaired coronary endothelial function may result in adverse clinical events because of the increased vascular and perivascular recruitment of neutrophils, macrophages, and platelets [119]. Taken together, these findings suggest that arteriolar changes, induced by ageing-related oxidative stress, impairs the vasoactive function of the coronary vessels in ageing.

### Kidney and skin

**Figure 5:**
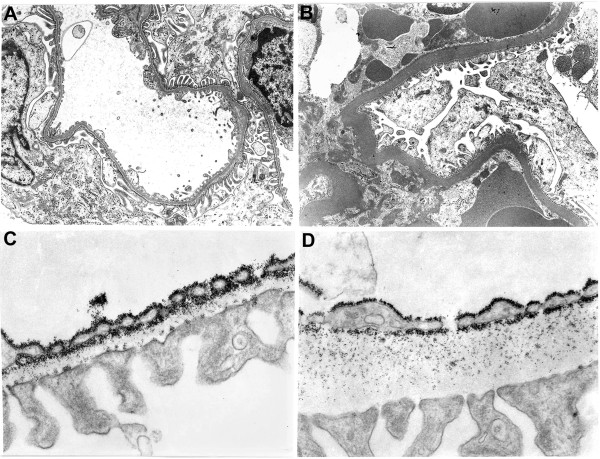
**Ultrastructural aspects of age-related changes in rat kidney microvessels.** Glomerular basement membrane of kidney in young **(A)** and old rat **(B)**, that shows the characteristic thickening of capillary wall. Magnification 5000×. Cationized ferritin distribution on glomerular basement membrane of young **(C)** and old rat kidney **(D)**. Magnification 30000×.

With ageing, a degenerative process occurs with the appearance of glomerular lesions, as a thickening of the glomerular basement membrane and Bowman’s capsule [120], parallel to glomerulosclerosis, interstitial fibrosis and progressive proteinuria [121]. Biochemical studies evidenced the age-related increase of collagen and decrease in glycosaminoglycans, particularly of heparan sulphate [122]. Ultrastructural studies, conducted in our laboratory, documented a marked thickening of the glomerular basement membrane in old rats (Figure 5A-B). In addition, young rats perfused with cationized ferritin in vivo showed a regular distribution of these molecules, along the internal and external lamina rara of the glomerular basement membrane (Figure 5C). In the old rats, ferritin was present only along the internal lamina rara (Figure 5D), suggesting that the age-related loss of anionic charged of heparan sulphate molecules is responsible for age-related proteinuria, also reported in human. In the kidney of aged rats, the glomerular and peritubular capillary loss correlates with alterations in VEGF and TSP-1 expression and also with the development of glomerulosclerosis and tubulointerstitial fibrosis, suggesting an impaired angiogenesis associated with progressive loss in renal microvasculature [62]. The mechanism of capillary loss in aged kidney has not been fully understood. Angiostatin is a potent inhibitor of angiogenesis in vivo. In aged rats angiostatin production is increased, as well as the activity of cathepsin D, the enzyme for angiostatin production [123]. In addition, NO availability is decreased and cathepsin D activated, suggesting a possible correlation between the increase of angiostatin production, capillary loss and interstitial damage in aged rat kidney [123]. NOS inhibition by L-NAME produced a stronger vasoconstriction in renal vessels of old compared with young rats [124,125], suggesting that endogenous NO production is necessary for the control of renal circulation. Moreover, post-mortem angiograms and histology studies, in elderly, showed wall thickening and narrowing of the vascular lumen of afferent arterioles, an alteration mainly depending on VSMC proliferation [126].

Tubulointerstitial fibrosis, in aged rats, was characterised by tubular injury and focal tubular cell proliferation, myofibroblast activation, macrophage infiltration with increased immunostaining for the adhesive proteins osteopontin and ICAM-1, and collagen IV deposition, as well as a decrease in eNOS expression in peritubular capillaries [127]. In addition, it has been reported that ageing induced oxidative stress in kidney and the attenuation of redox status can ameliorate microvascular function [128]. Renal oxidative stress was associated with an increase in ONOO^−^, NO and ROS levels, as well as iNOS activity [129]. Treatment with an antioxidant reduced the age-related renal dysfunction [129]. Moreover, in aged rats, NF-κB activation has been reported to contribute to the accumulation of oxidative stress [130].

**Figure 6:**
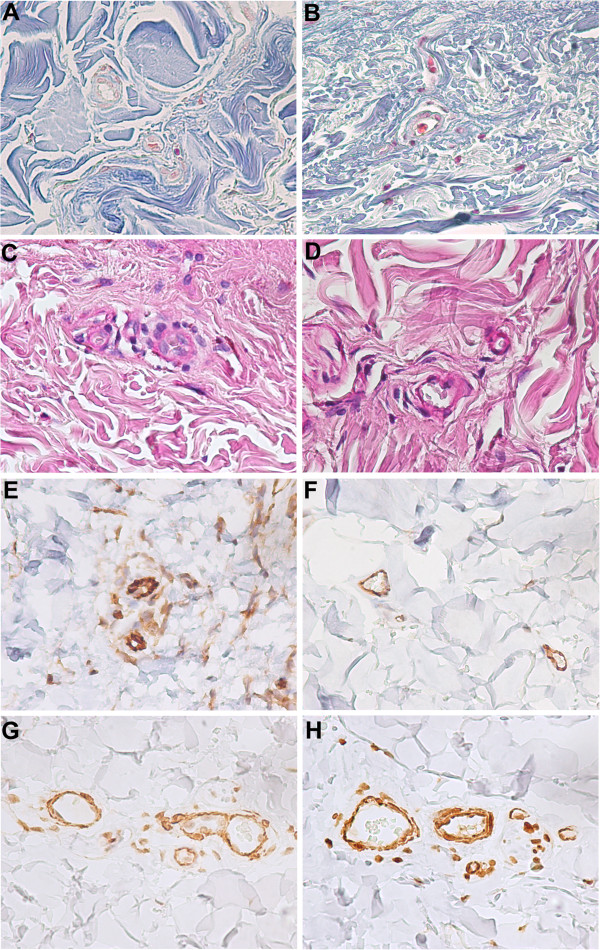
**Ageing in skin microcirculation.** Histology studies on paraffin-embedded sections (5 μm thick) of formalin-fixed skin of healthy subjects. Masson's trichrome staining shows collagen distribution (blue staining), around microvessels, in young **(A)** and old dermal skin **(B)**. PAS staining shows hyaline deposits (pink staining), around microvessels, in young **(C)** and old dermal skin **(D)**. CD31 immunostaining of young **(E)** and old dermal skin **(F)** showing the descrease of capillaries associated with ageing process. α-SMA immunostaining of young **(G)** and old dermal skin **(H)** showing the proliferation of VSMCs around aged microvessels. Magnification 40×.

Structural and functional alterations of the skin during the ageing process are due to some complex mechanisms, determined by intrinsic and extrinsic factors, which act synergistically [131]. Collagen fibers become thinner and change their aspect; in the deep dermis they become more fibrous. Thickened microvessels can be recognised by the increased intensity of the vascular PAS positive-diastase resistant staining, and by the perivascular collagen deposition (Figure 6). Elastic fibers show the tendency of fragmentation, with a pathological assembly [131,132]. With ageing, a progressive reduction of dermis vasculature is present, due to a reduction in the number and size of vascular vessels [131]. Age-related decrease in the number of dermal blood vessels is suggested to be due to an impairment of VEGF signalling [133]. In addition, it has been reported that eNOS activity is required for full expression of reflex cutaneous vasodilation, and its impairment in aged skin is associated with alterations in NO signalling [134], increase of oxidative stress and upregulation of arginase [135].

### Therapeutic targeting of microvascular ageing

Being assumed that microvascular dysfunction plays a key role in age-related end-organ failure, several therapeutic attempts have been suggested. We summarised the most diffuse anti-atherosclerotic and anti-ischemic treatments and more anti-ageing innovative strategies.

### Changes of lifestyle, anti-atherosclerotic and anti-ischemic treatments

Due to a high burden of cardiac risk factors and coronary atherosclerosis in subjects with angina and no obstructive coronary artery disease, lifestyle changes to modify risk factors are fundamental [136,137]. Cardiac rehabilitation is recommended for those patients who have limited physical activity; increased exercise capacity is related to the amelioration of atherosclerotic disease symptoms [138]. Statins may improve endothelial function by lipid-independent anti-inflammatory and antioxidant properties and the capacity to restore microvascular NO availability [139]. Angiotensin-converting enzyme inhibitors as well as angiotensin-renin blockers [140] have been shown to improve endothelium-dependent relaxation of coronary arteries by increasing NO availability [141]. Upregulation of arginase has emerged as an important factor contributing to reduce NO production by competing with endothelial NO synthase for the common precursor substrate L-arginine [142]. Arginase inhibitors may induce long-term improvement of microvascular function and limitation of myocardial injury following ischaemia–reperfusion [143].

### Antioxidant therapy

Some works focused the attention on antioxidant agents that can prevent or reduce the progression of end-organ microvascular dysfunction [144]. Antioxidants and free radical scavengers such as N-acetyl-cysteine (NAC), ascorbic acid and Propionyl-L-carnitine (PLC) showed a clinical efficacy in patients with endothelial dysfunction [145-149]. NAC, a derivative of cysteine, and ascorbic acid induced beneficial effects on oxidative stress and vascular dysfunction [145-147]. PLC is an ester of L-carnitine, that is required for the transport of fatty acids into the mitochondria [150]. PLC has been reported to modulate NF-kB activity in vascular cells [151] and to reduce age-related microvascular dysfunction and myocardial remodelling, including adhesion molecule expression [152]. In addition, it has been reported that PLC counteracts membrane lipid peroxidation and reduces post-ischemic endothelial dysfunction [153,154].

Ascorbate is essential for normal endothelial function [155] and prevents microvascular dysfunction and H2O2-mediated injury in cultured microvascular endothelial cells [144]. Other natural substances, such as aged garlic extract and resveratrol, have been documented to minimise oxidative stress and to stimulate endothelial NO generation, suggesting that antioxidant regimens can be efficacy to counteract adverse clinical effects of age-related microvascular endothelial dysfunction [74,75,156]. In *vitro* studies suggest that the molecular mechanisms of resveratrol-mediated vasoprotection involve NF-kB inhibition, upregulation of eNOS and antioxidant enzyme levels, and the prevention of oxidative stress–induced apoptosis [157,158]. Resveratrol supplementation may confer a significant vasoprotection in elderly humans [63].

### Novel anti-inflammatory therapies

Vascular ageing is associated with deregulation of TNF-α expression [36,159]. TNF-α is a master regulator of vascular inflammatory cytokines, chemokines and adhesion molecules. TNF-α plasma level increases with ageing and correlates with morbidity and mortality in the elderly patients [160,161]. Consequently an anti-TNF-α treatment (i.e., with etanercept, which binds and inactivates TNF-α) may exert vasoprotective effects, including a reduction of endothelial cell apoptosis and the downregulation of NAD(P)H oxidases activity [162]. Pharmacological inhibition of the poly(ADP-ribose) polymerase (PARP) pathway also represents a novel therapeutic target to improve ageing-associated cardiovascular dysfunction [163].

## Conclusions

Ageing elicits several structural and functional changes in the microvasculature. Reactive oxygen species and the concomitant oxidative and nitrosative stress play an important role in the process of ageing-related microvascular dysfunction, affecting vascular function as well as signalling transduction and gene expression. Although a significant progress has been achieved in describing the intrinsic age-related alterations of microvascular function, the age-related decline in endogenous antioxidant mechanisms, angiogenesis, endothelium-dependent vasodilation and microvascular permeability remains to be fully assessed. Increased knowledge may lead to new therapies targeting microvascular dysfunction and to improve clinical outcome. A key observation is that new therapeutic opportunities aimed to favour microvascular function are also associated with ameliorated organ function. An appropriate control of ageing process, in particular of oxidative stress, can clarify the efficacy of many pharmacological or nutritional approaches in order to delay the onset of age-dependent microvascular disease.

## Competing interests

The authors declare that they have no competing interests

## Authors’ contributions

MGS, AB, GA: writing of the manuscript; AF: revision of the manuscript; AO: financial support, administrative support, writing and final approval of the manuscript. All authors read and approved the final manuscript.
